# Stage 1 Registered Report: Anomalous perception in a Ganzfeld condition - A meta-analysis of more than 40 years investigation

**DOI:** 10.12688/f1000research.24868.3

**Published:** 2021-02-26

**Authors:** Patrizio E. Tressoldi, Lance Storm

**Affiliations:** 1 Science of Consciousness Research Group, Università degli studi di Padova, Padova, ITALY, 35131, Italy; 2 School of Psychology, University of Adelaide, Adelaide, Australia, 5005, Australia

**Keywords:** meta-analysis, ganzfeld; anomalous cognition, publication bias; consciousness

## Abstract

This meta-analysis is an investigation into anomalous perception (i.e., conscious identification of information without any conventional sensorial means). The technique used for eliciting an effect is the ganzfeld condition (a form of sensory homogenization that eliminates distracting peripheral noise). The database consists of peer-reviewed studies published between January 1974 and June 2020 inclusive. The overall effect size will be estimated using a frequentist and a Bayesian random-effect model. Moderators analyses will be used to examine the influence of level of experience of participants, the type of task and the peer-review level. Publication bias will be estimated by using four different tests. Trend analysis will be conducted with a cumulative meta-analysis and a meta-regression model with Year of publication as covariate.

## Introduction

The possibility of identifying pictures or video clips without conventional (sensorial) means, in a ganzfeld environment, is a decades old controversy, dating back to the pioneering investigation of Charles Honorton, William Braud and Adrian Parker between 1974 and 1975 ( Parker, 2017).

In the ganzfeld, a German term meaning ‘whole field’, participants are immersed in an homogeneous sensorial field were peripheral visual information is masked out by red light diffused by translucent hemispheres (often split halves of ping-pong balls or special glasses) placed over the eyes, while a relaxing rhythmic sound, or white or pink noise, is fed through headphones to shield out peripheral auditory information. Once participants are sensorially isolated from external visual and auditory stimulation, they are in a favourable condition for producing inner mental contents about a randomly selected target hidden amongst decoys. The mentation they produce can either be used by the participant to guide his/her target selection, or it can be used to assist in an independent judging process.

In the prototypical procedure, participants are tested in a room isolated from external sounds and visual information. After they made themselves comfortable in a reclining armchair, they receive the instructions related their task during the ganzfeld condition. Even if there are different verbatim versions, the instructions describe what they should do mentally in order to detect the information related to the target and how to filter out the mental contents not related to it. This information will be described aloud and recorded for playback before or during the target identification phase. After the relaxation phase, they are exposed to the ganzfeld condition for a period ranging from 15 to 30 minutes. During this phase, participants describe verbally all images, feelings, emotions, they deem related to the target usually a picture or a short videoclip of real objects or events.

Once the ganzfeld phase is completed, participants are presented with different choices (e.g. the target plus three decoys) of the same format, e.g. picture or videoclip, and they must choose which one is the target (binary decision). Alternatively, they may be asked to rate all four (e.g., from 0 to 100), to indicate the strength of relationship between the information detected during the ganzfeld phase and the images or video clips contents.

A variant of the judgment phase is to send the recording of the information retrieved during the ganzfeld phase to an external judge for independent ratings of the target. In order to prevent voluntary or involuntary leakage of information about the target by the experimenters, the research assistant who interact with the participants must be blind to the target identity until the participants’ rating task is over. The choice of the target and the decoys is usually made using automatic random procedures, and scores are automatically fed onto a scoring sheet.

There are three different ganzfeld conditions:

- Type 1: the target is chosen after the judgment phase;
- Type 2: the target is chosen before the ganzfeld phase;
- Type 3: the target is chosen before the ganzfeld phase and presented to a partner of the participant isolated in a separate and distant room. From an historical perspective, this last type is considered the typical condition.

These differences are related to some theoretical and perceptual concepts we will discuss later. It is important to note that type of task makes no difference to the participant who only engages in target identification *after* the ganzfeld phase.

## Review of the Ganzfeld Meta-Analyses

It is interesting to note that most of the cumulative findings (meta-analyses) of this line of investigation were periodically published in the mainstream journal *Psychological Bulletin*.

Honorton (1985) undertook one of the first meta-analyses of the many ganzfeld studies completed by the mid-1980s. In total, 28 studies yielded a collective hit rate (correct identification) of 38%, where mean chance expectation (MCE) was 25%. Various flaws in his approach were pointed out by Hyman (1985), but in their joint-communiqué they agree that “there is an overall significant effect in this database that cannot reasonably be explained by selective reporting or multiple analysis” ( Hyman & Honorton, 1986, p. 351).

A second major meta-analysis on a set of ‘autoganzfeld’ studies was performed by Bem & Honorton (1994). These studies followed the guidelines laid down by Hyman & Honorton (1986). Moreover the autoganzfeld procedure avoids methodological flaws by using a computer-controlled target randomization, selection, and judging technique. They overall reported hit rate of 32.2% exceeded again the mean chance expectation.

Milton & Wiseman (1999) meta-analysed further 30 studies collected for the period 1987 to 1997; reporting an overall nonsignificant standardized effect size of 0.013. However, Jessica Utts (personal communication, December 11, 2009) using the exact binomial test on trial counts only ( *N* = 1198; Hits = 327), found a significant hit rate of 27% ( *p* = 0.036).

Storm & Ertel (2001) comparing Milton & Wiseman’s (1999) database with Bem & Honorton’s (1994) one, found the two did not differ significantly. Furthermore Storm and Ertel went on to compile a 79-study database, which had a statistically significant average standardized effect size of 0.138.

Storm *et al.* (2010), meta-analysed a database of 29 ganzfeld studies published during the period 1997 to 2008, yielding an average standardized effect size of 0.14. Rouder *et al.* (2013) reassessing Storm *et al.*’s (2010) meta-analysis, with a Bayesian meta-analysis found evidence for the existence of an anomalous perception in the original dataset observing a Bayes Factor of 330 in support to the alternative hypothesis (p. 241). However, they contended the effect could be due to “difficulties in randomization” (p. 241), arguing that ganzfeld studies with computerized randomization had smaller effects than those with manual randomization. The reanalysis by Storm *et al.*’s (2013) showed that this conclusion was unconvincing as it was based on Rouder *et al.*’s faulty inclusion of different categories of study.

In the last meta-analysis by Storm & Tressoldi (2020), related to the studies published from 2008 to 2018, the average standardized effect size was 0.133; 95%CI: 0.06 - 0.18.

## This study

The main aim of this study is to meta-analyse all available ganzfeld studies dating from 1974 up to June 2020 in order to assess the average effect size of the database with the more advanced statistical procedures that should overcome the limitations of the previous meta-analyses. Furthermore, we aim to identify whether there are moderator variables that affect task performance. In particular, we hypothesize that participant type and type of task are two major moderators of effect size (see Methods section).

## Methods

### Reporting guidelines

This study will follow the guidelines of the APA Meta-Analysis Reporting Standard ( Appelbaum *et al.*, 2018) and the Preferred Reporting Items for Systematic Review and Meta-Analysis Protocols (PRISMA-P, Moher *et al.*, 2015).

### Studies retrieval

Retrieval of studies related to anomalous perception in a Ganzfeld environment is simplified, firstly by the fact that most of these studies have already been retrieved for previous meta-analyses, as cited in the introduction. Secondly, this line of investigation is carried out by a small community of researchers. Thirdly, most of the studies of interest to us are published in specialized journals that adopted the editorial policy of accepting paper with results that are statistically non-significant (according to the frequentist approach). This last condition is particularly relevant because it reduces the publication bias due to non publication (file drawer effect) of studies with statistically non significant results often as a consequence of a reduced statistical power.

Furthermore, in order to integrate the previous retrieval method, we will carry-out an online search with Google Scholar, PubMed and Scopus databases of all papers from 1974 to 2020 including in the title and/or the abstract the word “ganzfeld” (e.g. for PubMed: Search: ganzfeld[Title/Abstract] Filters: from 1974 – 2020).

### Studies inclusion criteria

The following inclusion criteria will be adopted:

- Studies related to anomalous perception in a ganzfeld environment;
- Studies must use human participants only (not animals);
- Number of participants must be in excess of two to avoid the inherent problems that are typical in case studies;
- Target selection must be randomized by using a Random Number Generator (RNG) in a computer or similar electronic device, or a table of random numbers. Randomization procedures must not be manipulated by the experimenter or participant;
- Studies must provide sufficient information (e.g., number of trials and outcomes) for the authors to calculate the direct hit-rates and effect size values, so that appropriate statistical tests can be conducted.
- Peer reviewed and not-peer reviewed studies, e.g. published in proceedings or doctoral dissertations.

### Variables coding

For each included study, one of the authors, expert in meta-analyses, will code the following variables:

- Authors;
- Year of publication;
- Number of trials;
- Number of hits;
- Number of choices of each trial;
- Task type (Type 1,2 or 3);
- Participants type (selected vs. unselected). The authors of the study will score as selected all participants that were screened for one or more particular characteristic deemed favourable for the performance in this type of task.
- Peer-Review level: level = 0 for studies published in conference proceedings; level = 1, for the studies published in scientific journals with full peer-review

The second author will independently check all studies, and the data will be compared with those extracted by the other author. Discrepancies will be corrected by inspecting the original papers.

The complete database will be made available through open access posting within the dedicated project in the Open Science Framework ( https://osf.io/t7sya/) platform.

### Effect size measures

As standardized measure of effect size, we will apply that used in Storm *et al.* (2010) and Storm & Tressoldi (2020) : Binomial Z score/√number of trials using the number of trials, the hits score and the chance probability as raw scores. The exact binomial Z score will be obtained applying the formula implemented online at http://vassarstats.net/binomialX.html. When this algorithm will not compute z when either number of trials or number of hits is low, we will use the one-tailed exact binomial p-value, to find the inverse normal z by using the online app at https://www.wolframalpha.com/widgets/gallery/view.jsp?id=540d8e149b5e7de92553fdd7b1093f6d

As standard error we will use the formula: √(hit rate * (1-hit rate)/trials * chance percentage *(1-chance percentage)).

In order to take in account the effect size overestimation bias in small samples, the effect sizes and their standard errors, will be transformed in the Hedge’s g effect sizes, with the corresponding standard errors by applying the formula presented in Borenstein *et al.* (2009, pp. 27–28: *g*=(1-(3/(4df-1)))* *d*).

### Overall effect size estimation

In order to take in account the between-studies heterogeneity, the overall effect size estimation of the whole database will be calculated by applying both a frequentist and a Bayesian random effect model for testing its robustness.

### Frequentist random-effect model

Following the recommendations of Langan *et al.* (2019), we will use the restricted maximum likelihood (REML) approach to estimate the heterogeneity variance with the Knapp and Hartung method for adjustment to the standard errors of the estimated coefficients ( Rubio-Aparicio *et al.*, 2018).

Furthermore, in order to control for possible influence of outliers, we will calculate the median and mode of the overall effect size applying the method suggested by Hartwig *et al.* (2020).

These calculations will be implemented in the R statistical environment with the metafor package v. 2.4 ( Viechtbauer, 2017). See syntax provided as extended data ( Tressoldi & Storm, 2020).

### Bayesian random-effect model

As priors for the overall effect size we will use a normal distribution with Mean = 0.01; *SD* =0.03, constrained positive, lower bound = 0 ( Haaf & Rouder, 2020), given our expectation of a positive value. As prior for the tau parameter we will use an inverse gamma distribution with shape = 1, scale = 0.15.

This Bayesian meta-analysis will be implemented with the MetaBMA package v. 0.6.3 ( Heck *et al.*, 2017).

### Publication bias tests

Following the suggestions of Carter *et al.* (2019), we will apply four tests to assess publication bias:

- the 3-parameter selection model (3PSM), as implemented by Coburn & Vevea (2019) with the package ‘ weightr’ v.2.0.2;
- the p-uniform* (star) v. 0.2.2 test as described by van Aert & van Assen (2019),
- the sensitivity analysis using the Mathur & VanderWeele (2020) package PublicationBias v.2.2.0.
- The Robust Bayesian meta-analysis test implemented with the RoBMA package v.1.0.5 ( Bartoš & Maier, 2020).

The three parameters model represent the average true underlying effect, *δ*; the heterogeneity of the random effect sizes, τ ^2^ and the probability that there is a nonsignificant effect in the pool of effect sizes. The probability parameter is modeled by a step function with a single cut point at *p* = 0.025 (one-tailed), which corresponds to a two-tailed *p* value of 0.05. This cut point divides the range of possible *p* values into two bins: significant and nonsignificant. The three parameters are estimated using maximum likelihood ( Carter *et al.*, 2019, p. 124).

The *p*-uniform* test, is an extension and improvement of the *p*-uniform method. P-uniform* improves upon *p*-uniform giving a more efficient estimator avoiding the overestimation of effect size in case of between-study variance in true effect sizes, thus enabling estimation and testing for the presence of between-study variance in true effect sizes. 

Sensitivity analysis as implemented by Mathur & VanderWeele (2020), assumes a publication process such that “statistically significant” results are more likely to be published than negative or “nonsignificant” results by an unknown ratio, *η* (eta). Using inverse-probability weighting and robust estimation that accommodates non-normal true effects, small meta-analyses, and clustering, it enables statements such as: “For publication bias to shift the observed point estimate to the null, ‘significant’ results would need to be at least 30-fold more likely to be published than negative or ‘nonsignificant’ results” (p. 1). Comparable statements can be made regarding shifting to a chosen non-null value or shifting the confidence interval.

The Robust Bayesian meta-analysis test is an extension of Bayesian meta-analysis obtained by adding selection models to account for publication bias. This allows model-averaging across a larger set of models, ones that assume publication bias and ones that do not. This test allows to quantify evidence for the absence of publication bias estimated with a Bayes Factor. In our case we will compare only two models, a random-effect model assuming no publication bias and a random-model assuming publication bias.

See Syntax Details in the Supporting Information

### Cumulative meta-analysis

In order to study the overall trend of the cumulative evidence we will perform a cumulative effect size estimation. Furthermore, we will estimate the overall effect size taking the variable “year of publication” as covariate using a meta-regression model.

### Moderators effects

We will compare the influence of the following three moderators: (i) Type of participant, (ii) Type of task and (iii) Level of peer-review.

As described in the Variable Coding paragraph, the variable Type of participant will be coded in a binary way: selected vs unselected. Type of task will be coded as Type 1, Type 2, and Type 3, as described in the Introduction and Level of Peer-review as 0 for studies published without a full peer-review or 1, for the studies published after a full peer-review.

### Statistical power

Once the overall effect size and its precision are estimated, we will calculate the number of trials necessary to achieve a statistical power of at least .80 with an α = .05. With this estimation we can examine how many studies in the database reached this threshold. The overall statistical power will be estimated with the R package metameta v.0.1.1. ( Quintana, 2020).

### Reporting

The search and selection of the studies will be presented by using a PRISMA flowchart.

### Descriptive statistics

Descriptive statistics will be produced related to the variables, trials, hits, participant type, and peer-review level task types.

### Overall effect size

We will present the estimated average effect size along with the corresponding 95% Confidence Intervals or Credible Intervals of both the frequentist and Bayesian random-model effect as described in the Methods section. We will calculate the values of τ ^2^ and I ^2^ ( Higgins & Thompson, 2002), and their confidence intervals, as measures of between-study variance.

### Publication bias tests

We will present the results of the four publication bias tests described in the Methods section.

### Cumulative effect size

The results of the cumulative meta-analysis will be represented with a cumulative forest plot.

### Moderator effects estimation and comparison

We will estimate and compare the average effect size along with the corresponding 95% Confidence Intervals of the two types the participant, the three task types and the two peer-review level, both with a parameter comparison of the overlap of their 95% CIs and with a focused hypothesis testing statistic e.g. ANOVA.

### Dissemination of information

Apart the Registered Report, all information related to this study will be made available open access at Open Science Framework https://osf.io/t7sya.

### Study status

The study has not started yet.

## Discussion

We will discuss the robustness of the overall results in order to determine a degree of confidence in the evidence for anomalous perception. In case of an insufficient degree of confidence in the evidence, we will consider whether it is worthwhile pursuing such a line of investigation and offer solutions to improve the evidence.

However, even if the overall results show a sufficient degree of evidence, we will discuss how this line of investigation can instil greater confidence by using a preregistration registry as proposed by Watt & Kennedy (2016) in order to reduce so-called questionable research practices ( John *et al.*, 2012), and provide more transparent procedures during data collection and analysis (see for example, the Transparent Psi Project; Kekecs *et al.*, 2019).

## Data availability

### Underlying data

No data are associated with this article

### Extended data

Figshare: Anomalous perception in a ganzfeld condition: a meta-analysis of more than 40 years of investigation. https://doi.org/10.6084/m9.figshare.12674618.v2 ( Tressoldi & Storm, 2020)

- Syntax Details.docx (Syntax related to all statistical analyses)

### Reporting guidelines

Figshare: PRISMA-P checklist for ‘Stage 1 Registered Report: Anomalous perception in a Ganzfeld condition - A meta-analysis of more than 40 years investigation’ https://doi.org/10.6084/m9.figshare.12674618.v2 ( Tressoldi & Storm, 2020) 

Data are available under the terms of the Creative Commons Attribution 4.0 International license (CC-BY 4.0).
