# Supplementary material for: Stage 1 Registered Report: Anomalous perception in a Ganzfeld condition - A meta-analysis of more than 40 years investigation
Source: F1000Res. 2021 Feb 26;9:826. Originally published 2020 Jul 30. [Version 3] doi: 10.12688/f1000research.24868.3 (PMC7883320; doi:10.12688/f1000research.24868.3)
Supplement: Peer review history

# Peer review: Reviewer response for version 3

**DOI:** 10.5256/f1000research.54788.r80300

**Published:** 2021-03-09

**Authors:** Stefan Schmidt

**Affiliations:** 1 Department of Psychosomatic Medicine and Psychotherapy, University of Freiburg, Freiburg, Germany; 2 Institute of Frontier Areas of Psychology and Mental Health, Freiburg, Germany

I am not satisfied with the revised version 3 in reply to my review. The authors have reacted to some of the minor issues that were have raised, but the reply regarding the two major issues and some of the other minor issues have not fully addressed my concerns.

**Major Issue 1: **

- The issue with the garbage in - garbage out problem was not addressed. The reply of the authors does not deal with the new problem of studies with likely poorer methodology before 1986.

**Major Issue 2: **

- The issue of using different effect sizes that belong to different classes of effect sizes is still pending. My plea for a short clarifying paragraph in the introduction was not taken up. Also, the results of prior meta-analyses are still not described by the same variables. I understand that these meta-analyses have used different approaches but I think it will be helpful to the reader if this is made explicit.

Regarding some of the procedures the authors apply, I do not see that they meet the criteria of specification. This refers to applying Google scholar as a research database. This database is not suitable since it is not transparent regarding content and also not regarding updates. The algorithms may even be influenced by cookies, IP addresses, etc. So if two people do the same research in this database we cannot guarantee to have the same results. The same situation is true regarding the webpage www.wolframalpha.com. Since you do not know how exactly this is operating or when it will change its mode, you cannot guarantee that you have transparently specified your procedures.

 I do not understand the answer of the authors regarding the issue of peer-review of proceedings. Does this mean proceedings are regarded as peer-reviewed or not?

 With respect to moderator comparison, the authors write: “…with a focused hypothesis testing statistic e.g. ANOVA.” I would be happy if this could be prespecified in an unambiguous matter.

Is the study design appropriate for the research question?

Partly

Have the authors pre-specified sufficient outcome-neutral tests for ensuring that the results obtained can test the stated hypotheses, including positive controls and quality checks?

Yes

Is the rationale for, and objectives of, the study clearly described?

Yes

Are sufficient details of the methods provided to allow replication by others?

Partly

Are the datasets clearly presented in a useable and accessible format?

Not applicable

Reviewer Expertise:

clinical and experimental reseach on mindfulness, medidation, consciousness and parapsychology

I confirm that I have read this submission and believe that I have an appropriate level of expertise to confirm that it is of an acceptable scientific standard, however I have significant reservations, as outlined above.

# Peer review: Reviewer response for version 2

**DOI:** 10.5256/f1000research.30185.r76033

**Published:** 2021-02-12

**Authors:** Stefan Schmidt

**Affiliations:** 1 Department of Psychosomatic Medicine and Psychotherapy, University of Freiburg, Freiburg, Germany; 2 Institute of Frontier Areas of Psychology and Mental Health, Freiburg, Germany

The present report describes a study protocol for conducting a meta-analysis on all Ganzfeld studies published so far. While there are many meta-analyses of Ganzfeld studies so far, this is the first one since 1985 that also includes the early studies from the beginning in 1974 to 1985. On the other end, the authors will include new studies from 2018 to 2020. The objective is to have for the first time the full ganzfeld database available in order to study moderators. This is a very sound aim and the resulting database will be of large value for future research.

 Many issues have been already raised by the two other reviewers and the authors have revised and improved the protocol accordingly.

 I have two major issues and some minor comments.

**1. Methodological Quality:**

 Reviewer J. Utts has already suggested including a rating for methodological quality. The author's reply stated that have already used a quality rating in earlier meta-analyses and have not found any correlation.

 Now regarding this study, the crucial difference with respect to earlier meta-analyses is that the authors also include the pre-communiqué studies before 1986. These studies have been already criticized for methodological quality and that is also the reason why they have not been included in earlier meta-analyses. Therefore, the planned meta-analysis may likely include studies with lower quality into the existing database. If these studies are given the same weight as studies with assumed higher quality, then the estimation of the aggregated effect size might be worse than before. This problem is known in the literature as “garbage in-garbage out”.

 Thus, I suggest to rate the study quality on a rating system that also codes for issues mentioned in the joint-communiqué from 1986.

 Just as an example: I have performed in 2002, a meta-analysis including all DMILS studies (direct mental interaction in living systems; a different experimental protocol in parapsychology)[ref1]. DMILS started at approx. the same time in the mid-1970s. I made a detailed rating of study quality and found that study quality was inversely related with effect-size. This resulted in excluding four weak studies and also in weighting the remaining studies in the meta-analysis according to their quality.

 This means that the authors will also need to pre-specify a procedure on how to deal with a likely correlation of study quality and effect size in the whole database and/or with a significant difference in study quality before 1986. The protocol needs to take care that the overall effect-size is not affected by studies with low quality or questionable procedures.

**2. Type of effect size:**

 There is some confusion with the type of effect size that is applied. Authors speak about “standardized effect size” or “mean standardized effect size” (e.g. page 4). Usually, all effect sizes are standardized, so this expression does not make much sense. There is the expression ‘standardized mean difference’ if one compares two means (not the case here) which refers to the fact that the difference is standardized to the standard deviation of the means. In principle, there are many different types of effect sizes depending on the kind of data that they are needed for. The ganzfeld case is not a standard case since here statistics is based on comparison to chance probabilities which is rather rare compared to other fields of science. Some researchers (e.g. Rosenthal) have grouped effect-sizes into families (d-type family, r-type family, etc.). This helps the reader to interpret the effect size (r ranges from -1 to +1, d-types can get larger than -1/+1, etc.). Also, rules of thump are usually given for the interpretation of the effect sizes of different families.

 Thus, it is suggested that the authors use consistent terminology throughout their protocol. This refers to their own effect-size computation (here it looks like they apply a d-type effect size since it can be transformed to Hedges’ g, which belongs to the d-type family), as well as to the description of earlier meta-analyses. In addition, I would be happy about a small paragraph in the introduction that explains on what different types of effect sizes have been used in the history of ganzfeld meta-analyses (e.g. Cohen’s h) how they relate to each other and why the effect size issue here is not a trivial one.

**Minor comments:**

 Page 3:

 Three types of Ganzfeld. These three types look like equivalent ones while they are in fact not from a historical perspective. From such a view type 3 would be the standard condition and the other ones special cases (no sender, target selected later). While this is of no importance for computation of the meta-analysis I suggest providing this information in order to make the publication more accessible for readers not familiar with parapsychology.

 In displaying other ganzfeld meta-analyses the description is inconsistent, sometimes hit rates and sometimes effect sizes are provided, sometimes p-values and sometimes confidence intervals. This should be streamlined, so the reader can compare the results. Maybe also a table would be of use?

 Page 4:

 I do not understand the sentence “…because it reduces the publication bias due to the non-rejection of the statistical null hypothesis often consequent to reduced statistical power. ” I have a slight idea what you want to express. Please clarify this, e.g. by making two sentences.

 Regarding databases for literature research please also include PsychInfo, and more important, Lexscien.

 With respect to study inclusion as well as variable coding a good standard is that this is done by two independent researchers. This should be also mentioned in the protocol.

 I am not entirely satisfied with the variable peer-review level. E.g. proceedings of the Parapsychological Associations are peer-reviewed. In the period before 2006 or 2008 there were full papers submitted and peer reviewed. This would be a different procedure than in the earlier or later times when only short proceedings were published.

 Page 5:

 Effect size calculation: the binominal distribution is approximated by the normal distribution. However, for small numbers, the exact binomial probability will be used. Please specify the cut-off for this procedure. Just referring to a web-site for this decision does not guarantee that others could replicate this procedure later on.

 Please provide the formula for the transformation into Heges’g instead of giving a reference.

 You are applying two aggregation models. Please specify how you would interpret your findings in case they diverge.

 Same for the four methods on publication bias estimation. What will be the interpretation if they result in different findings?

 “The Robust Bayesian meta-analysis test” on the lower part of the page should not be a headline.

 Page 6:

 Please specify your methodological approach on how to test for incline or decline effect. Or is the following sentence starting with “Furthermore,…” this description?

 Moderator effects (left column): Study quality needs to be assessed as moderator (see above).

 Moderator effects (right column): please specify the tests for the three (four) moderator effects.

 Extended data:

 There is a spelling error in the word ‘ganzfeld’

Is the study design appropriate for the research question?

Partly

Have the authors pre-specified sufficient outcome-neutral tests for ensuring that the results obtained can test the stated hypotheses, including positive controls and quality checks?

Yes

Is the rationale for, and objectives of, the study clearly described?

Yes

Are sufficient details of the methods provided to allow replication by others?

Partly

Are the datasets clearly presented in a useable and accessible format?

Not applicable

Reviewer Expertise:

clinical and experimental reseach on mindfulness, medidation, consciousness and parapsychology

I confirm that I have read this submission and believe that I have an appropriate level of expertise to confirm that it is of an acceptable scientific standard, however I have significant reservations, as outlined above.

## References

[1] : Distant intentionality and the feeling of being stared at: two meta-analyses. *Br J Psychol*.2004;95(Pt 2) : 10.1348/000712604773952449 235-47 10.1348/000712604773952449 15142304

# Response

**Published:** 2021-02-17

**Authors:** Patrizio Tressoldi

**Affiliations:** Dipartimento di Psicologia Generale, Università di Padova, Italy

**1. Methodological Quality:**

**......**Now regarding this study, the crucial difference with respect to earlier meta-analyses is that the authors also include the pre-communiqué studies before 1986. These studies have been already criticized for methodological quality and that is also the reason why they have not been included in earlier meta-analyses. Therefore, the planned meta-analysis may likely include studies with lower quality into the existing database.

 ..............This means that the authors will also need to pre-specify a procedure on how to deal with a likely correlation of study quality and effect size in the whole database and/or with a significant difference in study quality before 1986. The protocol needs to take care that the overall effect-size is not affected by studies with low quality or questionable procedures.

**Reply: As replied to Jessica Utts’, comments, in two previous meta-anaslyses (Storm, Tressoldi & Di Risio, 2010; Storm & Tressoldi, 2020) we didn’t find a statistical significant correlation between study quality assessed with an ad hoc system and effect size. We then plan to use the classical peer-review level as a conventional measure of studies quality. Furthermore, with our planned cumulative meta-analysis and regression meta-analysis with the variable Year of publication as covariate, it will be possible to examine the influence of the old studies on the overall effect size.**

****

**2. Type of effect size:**

**.............**There is some confusion with the type of effect size that is applied. Authors speak about “standardized effect size” or “mean standardized effect size” .

 ...........Thus, it is suggested that the authors use consistent terminology throughout their protocol. This refers to their own effect-size computation (here it looks like they apply a d-type effect size since it can be transformed to Hedges’ g, which belongs to the d-type family), as well as to the description of earlier meta-analyses.

 .........I would be happy about a small paragraph in the introduction that explains on what different types of effect sizes have been used in the history of ganzfeld meta-analyses (e.g. Cohen’s h) how they relate to each other and why the effect size issue here is not a trivial one.

**Reply: The type of effect size that we will use is described in the “Effect size measures” paragraph. In the introduction, we now used the term “average effect size”, when related to the overall results of the different meta-analyses. We also have clarified what raw data are used to compute the effect size.**

****

**Minor comments:**

 Page 3:

 Three types of Ganzfeld. These three types look like equivalent ones while they are in fact not from a historical perspective. From such a view type 3 would be the standard condition and the other ones special cases (no sender, target selected later). While this is of no importance for computation of the meta-analysis I suggest providing this information in order to make the publication more accessible for readers not familiar with parapsychology.

**Reply: On pag. 3, in the description of the three different ganzfeld conditions, we added  “From an historical perspective, this last type is considered the typical condition.**

 In displaying other ganzfeld meta-analyses the description is inconsistent, sometimes hit rates and sometimes effect sizes are provided, sometimes p-values and sometimes confidence intervals. This should be streamlined, so the reader can compare the results. Maybe also a table would be of use?

**Reply: Unfortunately, the average effect size were estimated with different methods and are not comparable.**

 Page 4:

 I do not understand the sentence “…because it reduces the publication bias due to the non-rejection of the statistical null hypothesis often consequent to reduced statistical power. ” I have a slight idea what you want to express. Please clarify this, e.g. by making two sentences.

**Reply: Now we changed the sentence as: “This last condition is particularly relevant because it reduces the publication bias due to non publication (file drawer effect) of studies with statistically non significant results often as a consequence of a reduced statistical power.”**

 Regarding databases for literature research please also include PsychInfo, and more important, Lexscien.

**Reply: Google Scholar includes all PsychInfo items. Lexscien is not open access and it does not allow a search with keywords.**

 With respect to study inclusion as well as variable coding a good standard is that this is done by two independent researchers. This should be also mentioned in the protocol.

**Reply: now we have specified “The second author will independently check all studies, and the data will be compared with those extracted by the other author”**

 I am not entirely satisfied with the variable peer-review level. E.g. proceedings of the Parapsychological Associations are peer-reviewed. In the period before 2006 or 2008 there were full papers submitted and peer reviewed. This would be a different procedure than in the earlier or later times when only short proceedings were published.

**Reply: As if only short proceeding were published, authors had to submit a full paper.**

 Page 5:

 Effect size calculation: the binominal distribution is approximated by the normal distribution. However, for small numbers, the exact binomial probability will be used. Please specify the cut-off for this procedure. Just referring to a web-site for this decision does not guarantee that others could replicate this procedure later on.

**Reply: Our binomial z score was always an exact binomial probability. Our description of how we obtained these values, makes our procedure replicable.**

 Please provide the formula for the transformation into Heges’g instead of giving a reference.

**Reply: now added.**

 You are applying two aggregation models. Please specify how you would interpret your findings in case they diverge.

**Reply: we suppose you are referring to the cumulative effect size and the regression model with Year of publication as covariate.** **Given that both methods are based on different algorithms, the divergence of their results will be commented according to their difference.**

 Same for the four methods on publication bias estimation. What will be the interpretation if they result in different findings?

**Reply: the four planned publication bias tests, are based on different algorithms, hence they different findings will be commented comparing their differences.**

 “The Robust Bayesian meta-analysis test” on the lower part of the page should not be a headline.

**Reply: Fixed**

 Page 6:

 Please specify your methodological approach on how to test for incline or decline effect. Or is the following sentence starting with “Furthermore,…” this description?

**Reply: we have rewritten this paragraph as “In order to study the overall trend of the cumulative evidence we will perform a cumulative effect size estimation. Furthermore, we will estimate the overall effect size taking the variable “year of publication” as covariate using a meta-regression model.”**

 Moderator effects (right column): please specify the tests for the three (four) moderator effects.

**Reply: the comparisons among the different moderators categories, are described in the paragraph “Moderator effects estimation and comparison”**

 Extended data:

 There is a spelling error in the word ‘ganzfeld’

**Reply: fixed, thank you.**

# Peer review: Reviewer response for version 1

**DOI:** 10.5256/f1000research.27439.r70878

**Published:** 2020-10-05

**Authors:** Julia Haaf

**Affiliations:** 1 Department of Psychology, Psychological Methods, University of Amsterdam, Amsterdam, The Netherlands

In this stage 1 registered report the authors describe a planned meta-analysis to target the Ganzfeld effect as is found in the parapsychological literature. Summarizing all conducted studies on the topic seemingly is a relevant research objective, though I might add that I do wonder about the quality of the conducted studies and their reporting. While the authors plan to conduct publication bias correction, to this point it is virtually impossible to fully account and correct for all the biases baked into the literature, let alone the parapsychological literature. I have a few comments on the statistical analysis, but some of these require some additional work by the authors.

  

1. **Effect size measure of interest.** The authors plan to report effect size measures based on the Binomial test. The binomial z-score seems like an appropriate choice given the models they plan to use. I wonder, however, why the authors decided to divide the binomial z-score by the square root of n, the sample size, given that the binomial z is calculated from the binomial mean and standard deviation, both dependent on n. In addition, if the binomial z corresponds to Fisher’s z then we know the standard error is 1/sqrt(n – 3). What is the standard error for z/sqrt(n), and how do we transform it to the standard error of Hedge’s g, as planned by the authors? Both frequentist and Bayesian meta-analysis requires the calculation of standard errors to weigh the study effects which is how meta-analysis accounts for sample size/precision. *This point must be addressed to make the article scientifically sound.*
2. **Random model or random-effects model.** The authors plan to use a model to account for between-study heterogeneity. In the meta-analytic literature, these models are called random-effects models (not random models). The wording of fixed-effect model vs. random-effects model from this literature is a bit unfortunate because it does not correspond to what is typically considered fixed vs. random effects in the statistical literature (Gelman, 2004, p. 20) ^1^. It might be better to describe the so-called random-effects model simply as a model accounting for between-study heterogeneity.
3. **Bayesian model with ordinal constraints.** The authors reference Rouder *et al.* (2019). I think this reference is perhaps misplaced. It does not really correspond to the sentence. Rouder *et al. *propose instead of interpreting the mean effect size across studies to focus on the distribution of true effect sizes. Therefore, the ordinal constraint is placed on each study’s true effect simultaneously. The way the authors describe it they plan to (only) apply an ordinal constraint on the overall effect. If the authors are interested in the question of whether all studies show an effect in the same direction, and I think this would be an interesting question for this application, I might shamelessly refer them to some of my recent work in this area (Haaf & Rouder, preprint) ^2^.
4. **Priors.** If the authors want to use a model that accounts for between-study heterogeneity (aka a random-effects model) they need to specify an additional prior distribution on that heterogeneity parameter. I would suggest adding this prior to this stage of the registered report. In the Haaf & Rouder preprint I mentioned above there are suggestions for priors on Fisher’s z that might be useful here. *This point must be addressed to make the article scientifically sound.*
5. **Which publication bias correction method is the best?** The authors plan to implement three ways of correcting for publication bias. If the three methods diverge in results, how will they interpret the results? Is there an ordering of method quality, or a way of combining them? Additionally, there have been newer development on publication bias corrections for Bayesian meta-analysis (Maier *et al.*, preprint) ^3^. Maybe this is also an option.
6. I really like the idea of a cumulative meta-analysis for this application! In Jasp (JASP Team, 2020) there is also an option to apply a cumulative Bayesian meta-analysis, maybe as a nice addition.

Is the study design appropriate for the research question?

Yes

Have the authors pre-specified sufficient outcome-neutral tests for ensuring that the results obtained can test the stated hypotheses, including positive controls and quality checks?

Partly

Is the rationale for, and objectives of, the study clearly described?

Yes

Are sufficient details of the methods provided to allow replication by others?

Partly

Are the datasets clearly presented in a useable and accessible format?

Not applicable

Reviewer Expertise:

Quantitative and mathematical psychology, expertise in Bayesian statistics, multilevel modeling and meta-analysis.

I confirm that I have read this submission and believe that I have an appropriate level of expertise to confirm that it is of an acceptable scientific standard, however I have significant reservations, as outlined above.

## References

[1] : Analysis of variance—why it is more important than ever. *The annals of statistics*.2005;33(1) :1-53

[2] : Does Every Study? Implementing Ordinal Constraint in Meta-Analysis.2020; 10.31234/osf.io/hf9se 10.31234/osf.io/hf9se 34807670

[3] : Robust Bayesian Meta-Analysis: Addressing Publication Bias with Model-Averaging.2020; 10.31234/osf.io/u4cns 10.31234/osf.io/u4cns 35588075

# Response

**Published:** 2020-10-10

**Authors:** Patrizio Tressoldi

**Affiliations:** Dipartimento di Psicologia Generale, Università di Padova, Italy

Thank you for your comments and suggestions. Here follows our replies;

1. **Effect size measure of interest.** The authors plan to report effect size measures based on the Binomial test. The binomial z-score seems like an appropriate choice given the models they plan to use. I wonder, however, why the authors decided to divide the binomial z-score by the square root of n, the sample size, given that the binomial z is calculated from the binomial mean and standard deviation, both dependent on n. In addition, if the binomial z corresponds to Fisher’s z then we know the standard error is 1/sqrt(n – 3). What is the standard error for z/sqrt(n), and how do we transform it to the standard error of Hedge’s g, as planned by the authors? Both frequentist and Bayesian meta-analysis requires the calculation of standard errors to weigh the study effects which is how meta-analysis accounts for sample size/precision. *This point must be addressed to make the article scientifically sound.*

**Reply:** **In the paragraph “Effect size measures” we have added how the effect sizes standard errors will be computed and how both will be transformed with the Hedges’s formulas**.

  

1. **Random model or random-effects model.** The authors plan to use a model to account for between-study heterogeneity. In the meta-analytic literature, these models are called random-effects models (not random models). The wording of fixed-effect model vs. random-effects model from this literature is a bit unfortunate because it does not correspond to what is typically considered fixed vs. random effects in the statistical literature (Gelman, 2004, p. 20) ^1^. It might be better to describe the so-called random-effects model simply as a model accounting for between-study heterogeneity.

**Reply: In the “Overall effect size estimation”, we have added this clarification**

  

1. **Bayesian model with ordinal constraints.** The authors reference Rouder *et al.* (2019). I think this reference is perhaps misplaced. It does not really correspond to the sentence. Rouder *et al. *propose instead of interpreting the mean effect size across studies to focus on the distribution of true effect sizes. Therefore, the ordinal constraint is placed on each study’s true effect simultaneously. The way the authors describe it they plan to (only) apply an ordinal constraint on the overall effect. If the authors are interested in the question of whether all studies show an effect in the same direction, and I think this would be an interesting question for this application, I might shamelessly refer them to some of my recent work in this area (Haaf & Rouder, preprint) ^2^.

**Reply: In the “Bayesian random-effect model” paragraph we have corrected this reference.**

  

1. **Priors.** If the authors want to use a model that accounts for between-study heterogeneity (aka a random-effects model) they need to specify an additional prior distribution on that heterogeneity parameter. I would suggest adding this prior to this stage of the registered report. In the Haaf & Rouder preprint I mentioned above there are suggestions for priors on Fisher’s z that might be useful here. *This point must be addressed to make the article scientifically sound.*

**Reply: In the “Bayesian random-effect model” paragraph we have added the tau parameter prior distribution (already available in the Syntax details file).**


**  **

1. **Which publication bias correction method is the best?** The authors plan to implement three ways of correcting for publication bias. If the three methods diverge in results, how will they interpret the results? Is there an ordering of method quality, or a way of combining them? Additionally, there have been newer development on publication bias corrections for Bayesian meta-analysis (Maier *et al.*, preprint) ^3^. Maybe this is also an option.

**Reply: The available literature hasn’t found yet the “best” publication bias for all conditions. We will analyse the robustness of our findings comparing the results of all the publication bias tests. As a fourth test, we will add the RoBMA test as suggested.**


**  **

1. I really like the idea of a cumulative meta-analysis for this application! In Jasp (JASP Team, 2020) there is also an option to apply a cumulative Bayesian meta-analysis, maybe as a nice addition.

**Reply: as a further test of the decline effect we will perform a meta-regression analysis using “Year of publication” as covariate (see “ Cumulative meta-analysis” paragraph).**

# Peer review: Reviewer response for version 1

**DOI:** 10.5256/f1000research.27439.r68427

**Published:** 2020-09-09

**Authors:** Jessica Utts

**Affiliations:** 1 Department of Statistics, University of California, Irvine, Irvine, CA, USA

This article outlines a meta-analysis the authors plan to conduct of all studies meeting certain criteria for the experimental realm in parapsychology called “ganzfeld.” The first ganzfeld experiments were conducted in the early 1970s. There have been multiple meta-analyses of ganzfeld studies over the years, but none have covered the entire research period, as the authors plan to do with this one. In addition to estimating an overall effect size, the proposed meta-analysis will examine two additional questions. One is whether the timing and/or participation of a “sender” makes a difference. This question will be examined by classifying the sessions into one of 3 types – target selected after the session, target selected before the session but with no sender, target selected before the session and a sender used. The other question of interest is whether there is a difference in effect size when the participant in the session was selected for characteristics thought to enhance performance.

 The authors are to be commended for addressing so many different issues that arise in meta-analysis, and for planning to use both frequentist and Bayesian methods. However, there are some details missing from the report that led to my answer of “partially provided” for the question about sufficient details to allow replication by others. Here are some details that are not provided as completely as needed in the paper if someone were to try to replicate their analyses. (It’s possible that they are provided in one of the references on protocols and reporting of meta-analysis, but not in the paper.)

- Will effect sizes be weighted by the size of the study? Obviously they should be. It makes no sense to give equal weight to a study with n = 20 and n = 100. But it isn’t clear in the methodology part of the report how the effect sizes will be combined.
- Will any measure of study quality be used? Some of the earlier studies were criticized for possible methodological problems. Or will studies that don’t meet certain quality criteria be omitted? Or is the plan to omit the studies that didn’t use proper randomization methods sufficient?
- Will studies that did not use standard targets (photographs, videos, locations) be excluded? For instance, at least one study used music instead of photographs or videos. Those probably should be excluded, because they represent possibly testing a different ability.
- The reference to using Hedges *g *to reduce bias for small studies is not clear. Hedge’s *g *is usually used for comparing means.
- It is not clear exactly what effect size measure will be used, but if I understand it correctly, it will be *z*/√n where *z *is found using the normal approximation to the binomial with continuity correction. Although that method gives results very close to using an exact binomial probability for sample sizes of perhaps 20 or more, it may not work well for small sample sizes. In fact the computation website mentioned in the report ( http://vassarstats.net/binomialX.html) won’t even compute *z* if either np or nq is less than 5. In such cases, an effect size could be found by using the exact binomial *p-*value, then finding the inverse normal *z* that gives that area in the upper tail. There is an effect size measure specially intended for proportions (Cohen’s *h*) but it may not be applicable if a study uses ratings instead of direct hits.
- It isn’t clear how the three types of studies will be compared. Will analysis of variance be used? Or, as mentioned, only looking at 95% confidence intervals for each type?  

Is the study design appropriate for the research question?

Yes

Have the authors pre-specified sufficient outcome-neutral tests for ensuring that the results obtained can test the stated hypotheses, including positive controls and quality checks?

Yes

Is the rationale for, and objectives of, the study clearly described?

Yes

Are sufficient details of the methods provided to allow replication by others?

Partly

Are the datasets clearly presented in a useable and accessible format?

Not applicable

Reviewer Expertise:

Statistical analysis and methods, with applications to various disciplines including parapsychology; statistics education.

I confirm that I have read this submission and believe that I have an appropriate level of expertise to confirm that it is of an acceptable scientific standard.

# Response

**Published:** 2020-10-10

**Authors:** Patrizio Tressoldi

**Affiliations:** Dipartimento di Psicologia Generale, Università di Padova, Italy

Thank you for your comments and suggestions. Here follows our replies:

- Will effect sizes be weighted by the size of the study? Obviously they should be. It makes no sense to give equal weight to a study with n = 20 and n = 100. But it isn’t clear in the methodology part of the report how the effect sizes will be combined.

**Reply: the random-effect model explained in the “Frequentist random-effect model” paragraph.  weights the studies by using the inverse of their variance plus an estimate of the heterogeneity of the studies τ ^2 ^; wi=1/(τ ^2^+vi)**


** **

- Will any measure of study quality be used? Some of the earlier studies were criticized for possible methodological problems. Or will studies that don’t meet certain quality criteria be omitted? Or is the plan to omit the studies that didn’t use proper randomization methods sufficient?

**Reply: In our 2010 and 2020 meta-analyses we assessed study quality using two judges whose ratings were highly correlated. We did not find a statistically significant correlation between study quality and ES. As to the proper randomization methods, our oldest studies applied the proper randomisation according to the Honorton-Hyman’s joint communiqué.**

**As a new way to test the correlation between study quality and ES we added a comparison between the studies published in journals with a full peer-review and the studies published in conference proceedings that usually have a less complete peer-review.**

 

- Will studies that did not use standard targets (photographs, videos, locations) be excluded? For instance, at least one study used music instead of photographs or videos. Those probably should be excluded, because they represent possibly testing a different ability.

**Reply: There are no theoretical reasons why targets different from images, pictures or video clips, cannot be used. We could assess whether their use will generate ES outliers.**

**We have assessed dynamic vs. static vs. objects/music in our 2020 study (no statitstical differences in ES). Objects/music category is a heterogeneous group, but ES for the single musical target study is not statistical different to the ES for objects.**


** **

- The reference to using Hedges *g *to reduce bias for small studies is not clear. Hedge’s *g *is usually used for comparing means.

**Reply: Hedges’ *g* can be applied to all continuous effect size like Cohen’s *d* independently from the experimental design (e.g. one and two-groups) see Borenstein et al (2009) pag. 30**


** **

- It is not clear exactly what effect size measure will be used, but if I understand it correctly, it will be *z*/√n where *z *is found using the normal approximation to the binomial with continuity correction. Although that method gives results very close to using an exact binomial probability for sample sizes of perhaps 20 or more, it may not work well for small sample sizes. In fact the computation website mentioned in the report ( http://vassarstats.net/binomialX.html) won’t even compute *z* if either np or nq is less than 5. In such cases, an effect size could be found by using the exact binomial *p-*value, then finding the inverse normal *z* that gives that area in the upper tail. There is an effect size measure specially intended for proportions (Cohen’s *h*) but it may not be applicable if a study uses ratings instead of direct hits.

**Reply: In the effect size measures paragraph, we added where that is the case, we will use wolframalpha calculator available online: https://www.wolframalpha.com/widgets/gallery/view.jsp?id=540d8e149b5e7de92553fdd7b1093f6d**


** **

- It isn’t clear how the three types of studies will be compared. Will analysis of variance be used? Or, as mentioned, only looking at 95% confidence intervals for each type?  

**Reply: I the “Moderator effects” paragraph we added we will compare the moderators effect** **by comparing the overlap of their 95% CIs and with a focused hypothesis testing statistic e.g. ANOVA.**
